# Supplementary material for: A mammalian-like piRNA pathway in Axolotl reveals the origins of piRNA-directed DNA methylation
Source: EMBO J. 2025 Nov 13;45(6):1933–56. doi: 10.1038/s44318-025-00631-w (PMC7618676; doi:10.1038/s44318-025-00631-w)
Supplement: Supplementary file 14 — Expanded View Figures [file 44318_2025_631_MOESM14_ESM.pdf]

Expanded View Figures

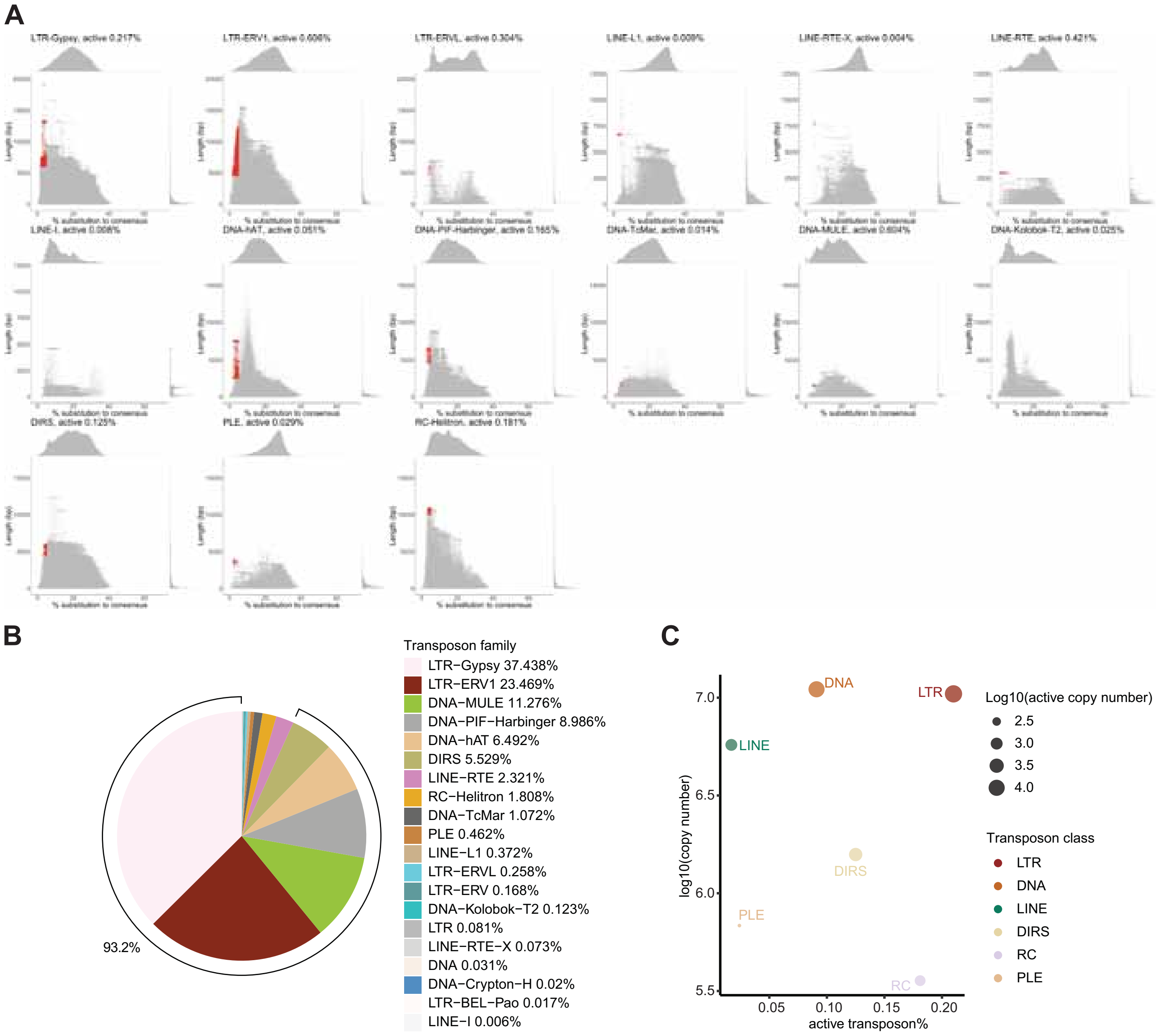

Figure EV1. Identification and distribution of potentially active transposons in the axolotl genome.

(**A**) Distribution of substitution rate and sequence length for every single copy in each transposon family. Defined potentially active copies are highlighted in red. (**B**) Composition of potentially active copies. (**C**) Scatter plot of percentage of potentially active copy number and total copy number for transposon classes. Transposon classes without potentially active copies are omitted.

 © The Author(s)

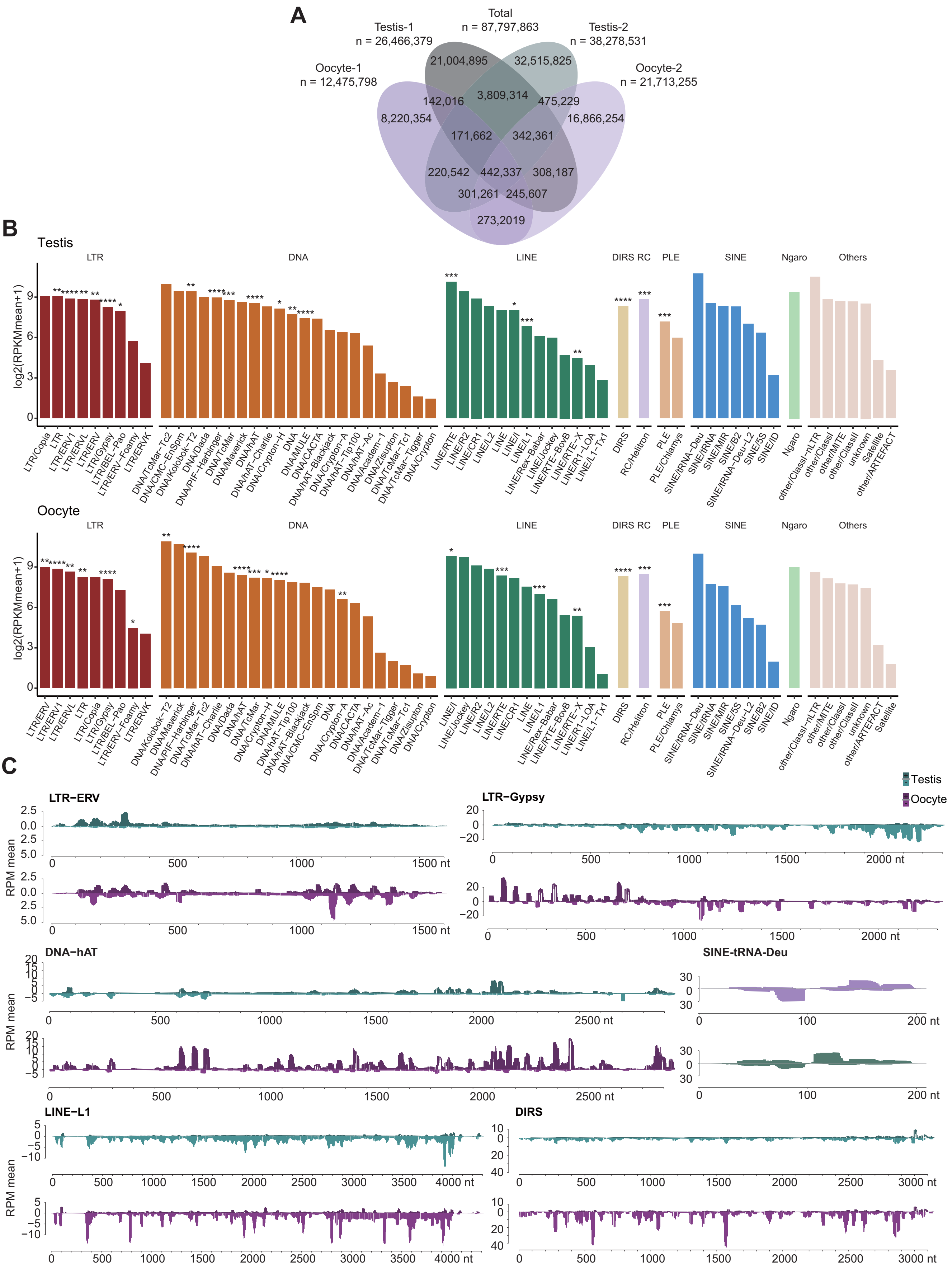
A
Total
n = 87,797,863
Testis-1
n = 26,466,379
Testis-2
n = 38,278,531
Oocyte-1
n = 12,475,798
Oocyte-2
n = 21,713,255
21,004,895
32,515,825
3,809,314
142,016
475,229
8,220,354
16,866,254
171,662
342,361
220,542
442,337
308,187
301,261
245,607
273,2019
B
Testis
LTR
DNA
LINE
DIRS
RC
PLE
SINE
Ngaro
Others
log2(RPKMmean+1)
LTR/Copia LTR LTR/ERV1 LTR/ERVL LTR/ERV LTR/Gypsy LTR/BEL-Pao LTR/ERV-Foamy LTR/ERVK
DNA/TcMar-Tc2 DNA/CMC-EnSpm DNA/Kolobok-T2 DNA/Dada DNA/PIF-Harbinger DNA/TcMar DNA/Maverick DNA/hAT DNA/hAT-Charlie DNA/Crypton-H DNA DNA/MULE DNA/CACTA DNA/hAT-Blackjack DNA/Crypton-A DNA/hAT-Tip100 DNA/hAT-Ac DNA/Academ-1 DNA/Zisupton DNA/TcMar-Tc1 DNA/TcMar-Tigger DNA/Crypton
LINE/RTE LINE/R2 LINE/CR1 LINE/L2 LINE LINE/I LINE/L1 LINE/Rex-Babar LINE/Jockey LINE/RTE-BovB LINE/RTE-X LINE/R1-LOA LINE/L1-Tx1
DIRS
RC/Helitron
PLE PLE/Chlamys
SINE/tRNA-Deu SINE/tRNA SINE/MIR SINE/B2 SINE/tRNA-Deu-L2 SINE/5S SINE/ID
Ngaro
other/ClassI-nLTR other/ClassI other/MITE other/ClassII unknown Satellite other/ARTEFACT
Oocyte
LTR
DNA
LINE
DIRS
RC
PLE
SINE
Ngaro
Others
log2(RPKMmean+1)
LTR/ERV LTR/ERV1 LTR/ERVL LTR LTR/Copia LTR/Gypsy LTR/BEL-Pao LTR/ERV-Foamy LTR/ERVK
DNA/Kolobok-T2 DNA/Maverick DNA/PIF-Harbinger DNA/TcMar-Tc2 DNA/hAT-Charlie DNA/Dada DNA/hAT DNA/TcMar DNA/Crypton-H DNA/MULE DNA/hAT-Tip100 DNA/hAT-Blackjack DNA/CMC-EnSpm DNA DNA/Crypton-A DNA/CACTA DNA/hAT-Ac DNA/Academ-1 DNA/TcMar-Tigger DNA/TcMar-Tc1 DNA/Zisupton DNA/Crypton
LINE/I LINE/Jockey LINE/R2 LINE/L2 LINE/RTE LINE/CR1 LINE LINE/L1 LINE/Rex-Babar LINE/RTE-BovB LINE/RTE-X LINE/R1-LOA LINE/L1-Tx1
DIRS
RC/Helitron
PLE PLE/Chlamys
SINE/tRNA-Deu SINE/tRNA SINE/MIR SINE/5S SINE/tRNA-Deu-L2 SINE/B2 SINE/ID
Ngaro
other/ClassI-nLTR other/MITE other/ClassII other/ClassI unknown other/ARTEFACT Satellite
C
Testis
Oocyte
LTR−ERV
LTR−Gypsy
DNA−hAT
SINE-tRNA-Deu
LINE−L1
DIRS
RPM mean
1500 nt
2000 nt
2500 nt
200 nt
4000 nt
3000 nt

◀ **Figure EV2. piRNA repertoires and transposon targeting in axolotl germlines.**

(**A**) The overlap of unique piRNA sequences among the four samples. (**B**) Mean piRNA signal level over testis or oocyte for each transposon family. ****, active copy number >1000; ***, active copy number >100; **, active copy number >10; *, active copy number >0. (**C**) Tracks for piRNAs targeting both sense and antisense strands of representative transposon consensus sequence. For all panels, testis, $n = 2$; oocyte, $n = 2$.

 © The Author(s)

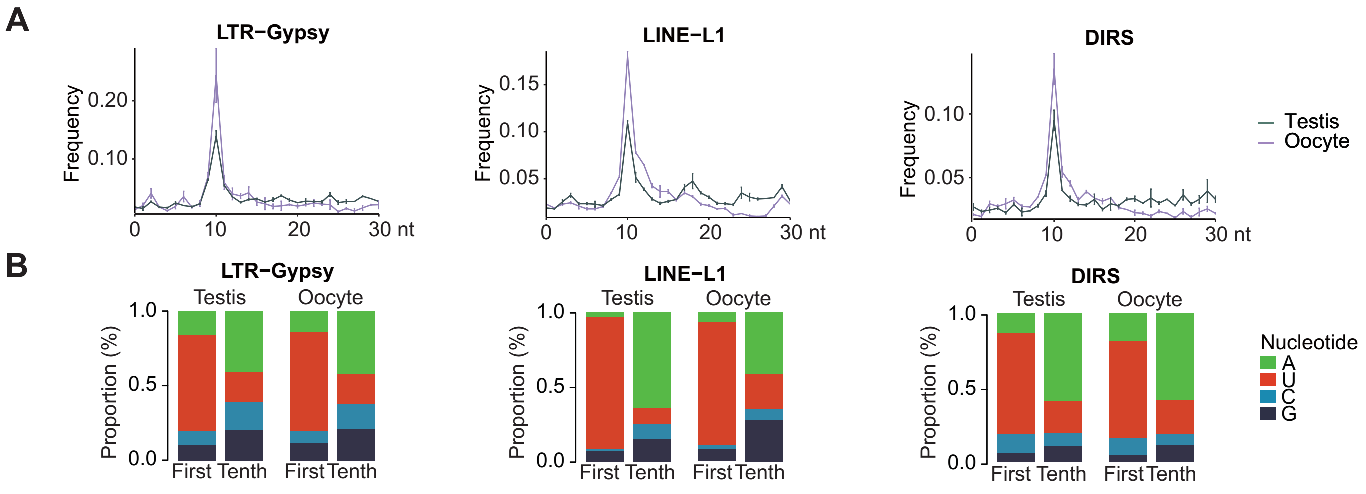

Figure EV3. Features of ping-pong cycle in axolotl germlines.

(**A**) Relative frequency of the nucleotide distance between 5′ ends of complementary piRNA pairs over representative transposon families. Mean and S.E.M. are presented. (**B**) Nucleotide composition of the first and tenth position in piRNAs over representative transposon families. A, Adenine; U, Uracil; C, Cytosine; G, Guanine. For all panels, testis, $n = 2$; oocyte, $n = 2$.

© The Author(s)

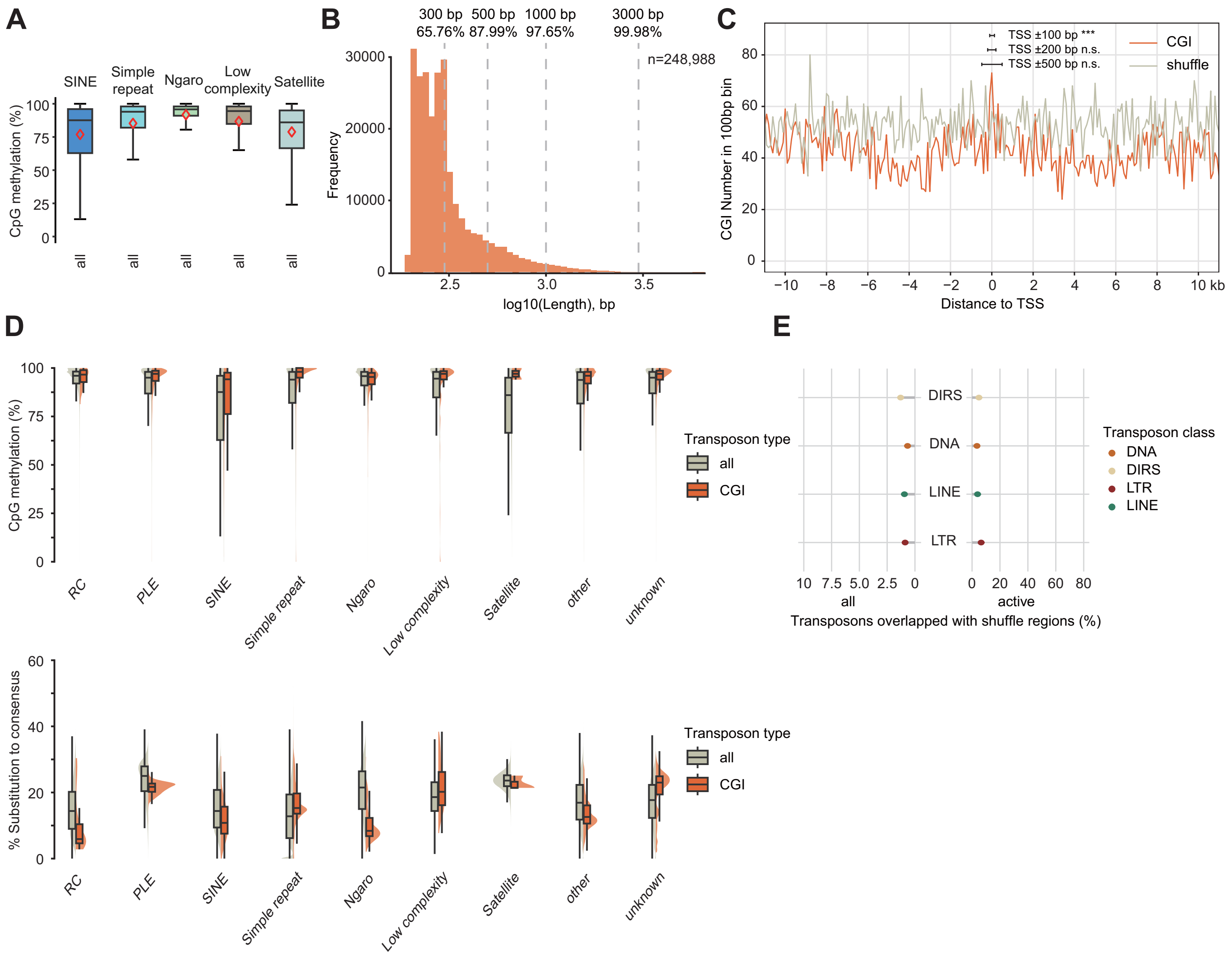

Figure EV4. DNA methylation landscape of minor transposon classes and CpG islands in axolotl spermatozoa.

(**A**) Percentages of CpG methylation levels over all copies of minor transposon classes in the axolotl. For boxplots, the middle line represents the median; boxes represent the 25th (bottom) and 75th (top) percentiles; whiskers represent median ± 1.5× interquartile range; and outside values are not shown. Rhombus, mean level. (**B**) Length distribution of all CGI regions ($n = 248,988$). (**C**) CGI enrichment over TSS and adjacent regions. For statistical tests, ****P* value < 0.001, n.s. not significant; Chi-square test. (**D**) Distribution of CpG methylation level and substitution rate for all transposon copies or copies overlapped with CGI of minor transposon classes. (**E**) Percentages of transposons overlapped with shuffled region. For all panels, spermatozoa, $n = 3$; pooled for analysis.

 © The Author(s)
