# Supplementary material for: A mammalian-like piRNA pathway in Axolotl reveals the origins of piRNA-directed DNA methylation
Source: EMBO J. Author manuscript; Available in PMC 2026 Jan 30. (PMC7618676; doi:10.1038/s44318-025-00631-w)
Supplement: Supplementary file 1 [file EMS211638-supplement-Reagent_table.docx]

**Structured Methods - Reagents and Tools Table**

*Instructions: Please complete the relevant fields below, adding rows as needed. The following page provides an example of a completed table and additional instruction for entering your data in the table.*

| **Reagent/Resource** | **Reference or Source** | **Identifier or Catalog Number** |
| --- | --- | --- |
|  |  |  |
| **Experimental Models** |  |  |
| White axolotl strain (d/d) | Elly M. Tanaka Lab | DD151 |
| **Recombinant DNA** |  |  |
|  |  |  |
|  |  |  |
| **Antibodies** |  |  |
|  |  |  |
|  |  |  |
| **Oligonucleotides and other sequence-based reagents** |  |  |
|  |  |  |
|  |  |  |
| **Chemicals, Enzymes and other reagents** |  |  |
| TRIzol | Thermo Fisher | 15596026 |
| T4 RNA Ligase 2 truncated KQ | New England Biolabs | M0373L |
| T4 RNA Ligase 1 | New England Biolabs | M0204L |
| SuperScript II | Thermo Fisher | 18064014 |
| Kapa polymerase | Kapa Biosystems | KK2602 |
| PmeI | New England Biolabs | R0560L |
| Monarch HMW DNA Extraction Kit | New England Biolabs | T3050L |
| NEBnext Enzymatic Methyl-seq kit | New England Biolabs | E7120L |
| **Software** |  |  |
| FastQC v0.11.8 | Babraham Bioinformatics |  |
| Cutadapt v2.9 | https://github.com/marcelm/cutadapt/ |  |
| Trim Galore v0.6.7 | Babraham Bioinformatics |  |
| STAR v2.7.0e | https://github.com/alexdobin/STAR/ |  |
| Bowtie2 v2.4.2 | http://bowtie-bio.sourceforge.net/bowtie2/index.shtml |  |
| SAMtools v1.9 | http://www.htslib.org/ |  |
| FeatureCounts v2.0.0 | http://subread.sourceforge.net/ |  |
| RepeatMasker v4.1.5 | http://www.repeatmasker.org/ |  |
| RepeatModeler v 2.0.5 | https://github.com/Dfam-consortium/RepeatModeler/ |  |
| DeepTE | https://github.com/LiLabAtVT/DeepTE/ |  |
| proTRAC v 2.4.3 | https://sourceforge.net/projects/protrac/ |  |
| circlize | https://github.com/jokergoo/circlize |  |
| gCluster | https://github.com/Xiangyang1984/Gcluster/ |  |
| Bismark v0.24.0 | https://www.bioinformatics.babraham.ac.uk/projects/bismark/ |  |
| SeqMonk v1.48.1 | Babraham Bioinformatics |  |
| ggplot2 v3.3.5 | https://sourceforge.net/projects/ggplot2.mirror/files/v3.3.5/ |  |
| RStudio v2022.07.0 | https://posit.co/download/rstudio-desktop/ |  |
| R v3.5.1 | https://www.r-project.org/ |  |
| Igv v2.3.72g | http://software.broadinstitute.org/software/igv/ |  |
| Illustrator software | Adobe Inc. |  |
| **Other** |  |  |
| Illumina HiSeq2500 | Illumina |  |
| Illumina NextSeq2000 | Illumina |  |
