# Supplementary material for: A mammalian-like piRNA pathway in Axolotl reveals the origins of piRNA-directed DNA methylation
Source: EMBO J. Author manuscript; Available in PMC 2026 Jan 30. (PMC7618676; doi:10.1038/s44318-025-00631-w)
Supplement: Supplementary file 2 [file EMS211638-supplement-Supplementary_file.docx]

**Supplementary Information**

## A mammalian-like piRNA pathway in Axolotl indicates the origins of piRNA-directed DNA methylation

## Authors:

## Xinyu Xiang (相欣雨)^1,2,3^, Anni Gao (高安妮)^3^, Dominik Handler^4^, Francisco Falcon^5,6^, Diego Rodriguez-Terrones^5^, Sergej Nowoshilow^5^, Wanlu Liu (刘琬璐)^3,7,8^, Elly M. Tanaka^5^, Dónal O’Carroll^1,2,9^

### Affiliations:

^1^Centre for Regenerative Medicine, Institute for Regeneration and Repair, Institute for Stem Cell Research, University of Edinburgh, 5 Little France Drive, Edinburgh, EH16 4UU, UK.

^2^Wellcome Centre for Cell Biology, University of Edinburgh, Michael Swann Building, Max Born Crescent, Edinburgh, EH9 3BF, UK.

^3^Zhejiang University-University of Edinburgh Institute (ZJU-UoE Institute), Zhejiang University School of Medicine, International Campus, Zhejiang University, 718 East Haizhou Rd., Haining, 314400, China.

^4^Institute of Molecular Biotechnology of the Austrian Academy of Sciences (IMBA), Vienna BioCenter (VBC), Campus Vienna Biocenter, Vienna, 1030, Austria.

^5^Institute of Molecular Pathology (IMP), Vienna Biocenter (VBC), Campus Vienna Biocenter, Vienna, 1030, Austria.

^6^Vienna BioCenter PhD Program, Doctoral School of the University of Vienna and Medical University of Vienna, Vienna, Austria.

^7^Department of Rheumatology and Immunology of the Second Affiliated Hospital, Zhejiang University School of Medicine, Zhejiang University, Hangzhou, 310009 China

^8^Dr. Li Dak Sum & Yip Yio Chin Center for Stem Cell and Regenerative Medicine, Zhejiang University, 866 Yuhangtang Rd., Hangzhou, 310058, China

^9^Leading author: Dónal O’Carroll ([donal.ocarroll@ed.ac.uk](mailto:donal.ocarroll@ed.ac.uk))

## Supplementary Figure 1. A Distribution of substitution rate and sequence length for every single copy in each transposon family. Defined potentially active copies are highlighted in red. B Composition of potentially active copies. C Scatter plot of percentage of potentially active copy number and total copy number for transposon classes. Transposon classes without potentially active copies are omitted.

## Supplementary Figure 2. A The overlap of unique piRNA sequences among the four samples. B Mean piRNA signal level over testis or oocyte for each transposon family. ****, active copy number > 1000; ***, active copy number > 100; **, active copy number > 10; *, active copy number > 0. C Tracks for piRNAs targeting both sense and antisense strands of representative transposon consensus sequence. For all panels, testis, n = 2; oocyte, n = 2.

## Supplementary Figure 3. A Relative frequency of the nucleotide distance between 5′ ends of complementary piRNA pairs over representative transposon families. Mean and S.E.M. are presented. B Nucleotide composition of the first and tenth position in piRNAs over representative transposon families. A, Adenine; U, Uracil; C, Cytosine; G, Guanine. For all panels, testis, n = 2; oocyte, n = 2.

##  

## Supplementary Figure 4. A Percentages of CpG methylation levels over all copies of minor transposon classes in the axolotl. For box plots, the middle line represents the median; boxes represent the 25th (bottom) and 75th (top) percentiles; whiskers represent median ± 1.5× interquartile range; and outside values are not shown. Rhombus, mean level. B Length distribution of all CGI regions (n=248,988). C CGI enrichment over TSS and adjacent regions. For statistical tests, ***P-value  <  0.001, n.s. not significant; Chi-square test. D Distribution of CpG methylation level and substitution rate for all transposon copies or copies overlapped with CGI of minor transposon classes. E Percentages of transposons overlapped with shuffled region. For all panels, spermatozoa, n = 3; pooled for analysis.
